# Rise and fall of total mesorectal excision with lateral pelvic lymphadenectomy for rectal cancer: an updated systematic review and meta-analysis of 11,366 patients

**DOI:** 10.1007/s00384-021-03946-2

**Published:** 2021-06-14

**Authors:** Gabriele Anania, Richard Justin Davies, Alberto Arezzo, Francesco Bagolini, Vito D’Andrea, Luigina Graziosi, Salomone Di Saverio, Georgi Popivanov, Isaac Cheruiyot, Roberto Cirocchi, Annibale Donini

**Affiliations:** 1 Dipartimento di Scienze Mediche, Università degli Studi di Ferrara, Ferrara, Italy; 2 Cambridge Colorectal Unit - Addenbrooke’s Hospital, Cambridge University Hospitals NHS Foundation Trust, Cambridge, UK; 3 Department of Surgical Sciences, University of Torino, Corso Dogliotti 14, 10126 Torino, Italy; 4 Department of Surgical Sciences, Sapienza University of Rome, Piazzale Aldo Moro 5, 00185 Rome, Italy; 5 Department of Surgery and Biomedical Sciences, University of Perugia, 06121 Perugia, Italy; 6 Department of General Surgery (S.D.S., G.I., E.Z., G.C.), University of Insubria, University Hospital of Varese, ASST Sette Laghi, Regione Lombardia, Italy; 7 Department of Surgery, Military Medical Academy, ul. “Sv. Georgi Sofiyski” 3, 1606 Sofia, Bulgaria; 8 Department of Human Anatomy, University of Nairobi, Nairobi, Kenya

**Keywords:** Rectal cancer, Total mesorectal excision, Lateral pelvic lymphadenectomy

## Abstract

**Abstract:**

The role of lateral lymph node dissection (LLND) during total mesorectal excision (TME) for rectal cancer is still controversial. Many reviews were published on prophylactic LLND in rectal cancer surgery, some biased by heterogeneity of overall associated treatments. The aim of this systematic review and meta-analysis is to perform a timeline analysis of different treatments associated to prophylactic LLND vs no-LLND during TME for rectal cancer.

**Methods:**

A literature search was performed in PubMed, SCOPUS and WOS for publications up to 1 September 2020. We considered RCTs and CCTs comparing oncologic and functional outcomes of TME with or without LLND in patients with rectal cancer.

**Results:**

Thirty-four included articles and 29 studies enrolled 11,606 patients. No difference in 5-year local recurrence (in every subgroup analysis including preoperative neoadjuvant chemoradiotherapy), 5-year distant and overall recurrence, 5-year overall survival and 5-year disease-free survival was found between LLND group and non LLND group. The analysis of post-operative functional outcomes reported hindered quality of life (urinary, evacuatory and sexual dysfunction) in LLND patients when compared to non LLND.

**Conclusion:**

Our publication does not demonstrate that TME with LLND has any oncological advantage when compared to TME alone, showing that with the advent of neoadjuvant therapy, the advantage of LLND is lost. In this review, the most important bias is the heterogeneous characteristics of patients, cancer staging, different neoadjuvant therapy, different radiotherapy techniques and fractionation used in different studies. Higher rate of functional post-operative complications does not support routinely use of LLND.

**Supplementary Information:**

The online version contains supplementary material available at 10.1007/s00384-021-03946-2.

## Introduction

Although commonly performed in urologic [1] and gynaecologic [2] surgery, the role of lateral lymph node dissection (LLND) is still a very controversial topic in rectal cancer treatment [3]. This procedure, reported in Japan in the 1970s [4, 5], was standardized by Moriya at the end of the 1980s: “On the basis of the extent of lateral node spread, two types of lateral node dissection were performed, consisting of preservation of internal iliac vessels (conventional) and en-bloc excision of these vessels (extended)” [6].

Currently, total mesorectal excision (TME) remains the gold standard for surgical treatment of mid and low rectal cancer. In contrast, the place of LLND remains a matter of controversy between Eastern and Western surgical guidelines [7–12]. The main conceptual difference is the fact that the lateral pelvic lymph nodes are considered as localized disease in Japanese clinical practice, whereas the West treats them as systemic disease [13–15]. For this reason, in Japan, prophylactic LLND is always performed in patients with stage II/III lower rectal cancer, whereas in the West, chemoradiotherapy (CRT) is routinely performed, thus generally avoiding a more invasive surgical approach [16].

To date, seven systematic reviews and meta-analyses have provided the highest levels of evidence to support the role of LLND for rectal cancer [17–23]. This new systematic review and meta-analysis aims to perform an updated analysis of the different types of treatments associated with prophylactic LLND vs. no-LLND (NLLND) in rectal cancer surgery.

## Methods

We performed a systematic review adhering to AMSTAR 2 principles [24]. A literature search was performed from two authors (R.C., F.B.) in PubMed, SCOPUS and WOS for publications up to 1 September 2020. The protocol for this study was registered on PROSPERO, a prospective international database for reviews under the registration number 42020186525.

### Inclusion criteria

We considered RCTs (randomized control trial) and CCTs (clinical control trials) comparing patients with rectal cancer who underwent rectal resection and TME with versus without LLND.

### Exclusion criteria

Patients having surgery without TME.

The Preferred Reporting Items for Systematic Reviews and Meta-analyses (PRISMA) guidelines were followed [25](ESM6). The keywords used for PubMed database research were: “extended lymphadenectomy,” “pelvic lymphadenectomy,” “lateral lymph-node dissection,” “total mesorectal excision,” “rectal resection,” “rectal cancer,” and their combinations. The search strategy performed on PubMed was the following: “extended lymphadenectomy”[All Fields] AND (“rectum”[MeSH Terms] OR rectum[All Fields]) “pelvic lymphadenectomy “[All Fields] AND (rectum[MeSH Terms] OR rectum[All Fields]) “lateral lymph-node dissection”[All Fields] AND (rectum[MeSH Terms] OR rectum[All Fields]).

We also manually searched the references of identified articles and relevant reviews and searched conference proceedings, theses and published abstracts on Google scholar. No language restriction was applied.

### Outcomes

The primary outcomes were the incidence of local recurrence and distant recurrence at 5 years. The secondary outcomes were the 5-year overall and disease-free survival and the incidence of urinary dysfunction (retention), urinary incontinence, evacuatory dysfunction and sexual dysfunction.

The assessment of methodological quality was performed independently by two authors (RC, CR). The risk of bias of randomized control trials (RCTs) was assessed using methods described in the Cochrane Handbook for Systematic Reviews of Interventions [26] and the ROBINS-I tool [27] for observational studies. In the ROBINS-I tool, risk of bias is assessed within specified domains, including bias due to confounding, bias in selection of participants into the study, bias in classification of interventions, bias due to deviations from intended interventions bias due to missing data, bias in measurement of outcomes, bias in selection of the reported result and overall bias. Bias assessments were tabulated with explanation. Disagreements were resolved via discussion between the investigators. Graphic representation of the results was produced using the Robvis online tool [28] (ESM4-5).

### Statistical analysis

This meta-analysis was conducted using the Review Manager (RevMan version 5.3.5) computer program (Copenhagen: The Nordic Cochrane Centre, The Cochrane Collaboration, 2014).

The dichotomous outcomes were pooled with a random-effects model with the Mantel-Haenszel method to estimate risk ratios (RRd) and their 95% confidence intervals [29]. Clinical heterogeneity was tested using τ2, Cochrane’s Q and I^2^ statistics. We considered an *I*^2^ value exceeding 50% to be indicative of heterogeneity [30].

We used a random-effect analysis model for the high clinical heterogeneity and statistically significant higher chi^2^ value and *I*^2^ [31]. In all remaining circumstances, we used the random-effects model.

The following subgroup analyses were performed to reduce the heterogeneity:

- LLND vs. NLLND
- LLND vs. NLLND and adjuvant therapy
- LLND and adjuvant therapy vs. NLLND and adjuvant therapy
- LLND vs nCRT and NLLND
- nCRT and LLND vs nCRT and NLLND

## Results

The PRISMA flow diagram for the systematic review is presented in SDC 1 (ESM1). The initial search yielded 2833 potentially relevant articles. After the removal of duplicates, 1767 studies underwent screening of titles/abstracts for relevance and assessment for eligibility; 1724 further articles were eventually excluded leaving 43 studies for analysis of the full text. Of these, nine studies, included in the other systematic review [17–23], were successively excluded (SDC 2)(ESM2) [5, 32–39]. The remaining 34 articles and 29 studies (11.606 patients: 5161 underwent LLND and 6445 NLLND) were included in this systematic review and meta-analysis. One study (Tsukamoto 2020) [40] overlapped with a previous study (Fujita 2017) [41]. In effect, the study of Tsukamoto et al. is the result of a long-term follow-up of the Japan Clinical Oncology Group (JCOG) 0212 (ClinicalTrials.gov NCT00190541) published previously from Fujita et al. in 2017. The other studies included as RCT [40–44] are all based on the same trial (JCOG0212) and therefore represent the same group of patients. The studies of Nagawa 2001 [45] and Watanabe 2002 [7] are both from the same single institution with overlapped years.

### Characteristics of the studies

The 28 included studies were published between 1994 and 2020; patients were enrolled between 1985 and 2016 (Table 1). In all studies, the cancer was located at the rectum, except one that also included patients with anal cancer [66]. The level of the cancer was reported in 22 studies. In 18 studies (85.7%%), the tumour was located in the lower rectum. A small proportion of studies included patients with upper rectal cancer (14.3%) [64, 67, 68].

**Table 1:** Included studies

	Author and year of publication	Nation	Type of study	Time of enrolment	Location of cancer	Clinical AJCC staging	Patients included	Type of rectal resection
1	Tsukamoto 2020 [40]	Japan	RCT	2003-2010	Rectum L	II/III	701	NR
2	Oki 2019 [46]	Japan	RCT	2006-2009	Rectum	I/II/III	445	RAR APR HP Others
3	Nishizaki 2019 [47]	Japan	Retrospective CCT	NR	Rectum	NR	155	NR
4	Ogura 2019 [48]	Australia//Korea/Netherlands/Japan/UK/USA	Prospective CCT	2009-2013	Rectum L	NR	968	RAR APR PE Others
5	Matsuda 2018 [49]	Japan	Retrospective CCT	2005-2016	Rectum	I/II/III	45	RAR APR HP Others
6	Park 2018 [50]	Korea	Retrospective CCT	2011-2016	Rectum L	II/III	361	RAR APR HP
7	Ito 2018 [43]	Japan	RCT	2003-2010	Rectum	NR	701	NR
8	Dev 2017 [51]	India	RCT	NR	Rectum L	II/III	240	NR
9	Georgiu 2017 [17]	UK	Retrospective CCT	2006-2009	Rectum	NR	38	PE
10	Ishihara 2017 [52]	Japan	Retrospective CCT	2003-2015	Rectum L	NR	222	RAR APR HP PE Others
11	Fujita 2017 [41]	Japan	RCT	2003-2010	Rectum L	II/III	701	RAR
12	Tamura 2017 [53]	Japan	Retrospective CCT	2000-2015	Rectum L	IV	50	NR
13	Kim 2017 [54]	Korea	Retrospective CCT	NR	Rectum L	NR	377	RAR APR
14	Ogura 2017 [48]	Japan	Retrospective CCT	2005-2014	Rectum L	II/III	363	RAR APR HP Others
15	Saito 2016 [44]	Japan	RCT	2003-2010	Rectum	II/III	701	NR
16	Ozawa 2016 [55]	Japan	Retrospective CCT	1995-2004	Rectum L	II/III	998	RAR APR Others
17	Akiyoshi 2019 [56]	Japan	Retrospective CCT	2004-2010	Rectum L	II-III	127	RAR APR HP Others
18	Fujita 2013 [42]	Japan	RCT	2003-2010	Rectum L	II/III	701	RAR
19	Akasu 2009 (79)	Japan	Retrospective CCT	1992-2006	Rectum L	NR	69	NR
20	Kusters 2009 [57]	Netherlands/ Japan	Prospective CCT	1993-2002	Rectum L	I/II/III	1.079	RAR APR HP PE
21	Kobayashi 2009 [58]	Japan	Retrospective CCT	1991-1998	Rectum L	I/II/III	1.272	NR
22	Shiozawa 2007 [59]	Japan	Retrospective CCT	1990-2000	Rectum L	NR	169	NR
23	Kim 2007 [60]	Korea	Retrospective CCT	1995-2000	Rectum L	III	290	RAR APR
24	Yano 2007 [61]	Japan	Prospective CCT	1995-2003	Rectum	I/II/III/IV	109	RAR APR HP
25	Kyo 2006 [62	Japan	Prospective CCT	1998-2000	Rectum	I/II/III/IV	37	RAR APR HP
26	Col 2005 [63]	Turkey	Retrospective CCT	1997-2000	Rectum	NR	170	RAR APR
27	Hasdemir 2005 [64]	Turkey	Retrospective CCT	NR	Rectum U/M/L	I/II/III	170	RAR APR
28	Matsuoka 2005 [65]	Japan	Prospective CCT	1998 - 2003	Rectum	NR	57	RAR
29	Fujita 2003 [66]	Japan	Retrospective CCT	1985-1998	Rectum L Anal Canal	II/III	246	RAR APR
30	Maeda 2003 [67]	Japan	Prospective CCT	1988-1996	Rectum U/L	NR	77	RAR APR
31	Watanabe 2002 [7]	Japan	Retrospective CCT	1985-1995	Rectum L	NR	115	RAR APR HP PE
32	Nagawa 2001 [45]	Japan	RCT	1993-1995	Rectum L	NR	45	RAR APR
33	Suzuki 1995 [68]	Japan	Retrospective CCT	1963-1990	Rectum U/L	NR	192	RAR APR Others
34	Moreira 1994 [69]	Japan	Retrospective CCT	1981-1991	Rectum	NR	178	NR

*APR* abdominoperineal resection, *IR* intersphincteric resection, *LLND* lateral lymph node dissection, *Non-LLND* non-lateral lymph node dissection, *PE* pelvic exenteration, *RAR* rectal anterior resection, *U* upper, *M* Mid, *L* lower, *ME* mesorectal excision

The clinical AJCC staging was reported in 17 studies (50%): II/III stages (9 studies), I/II/III stages (4 studies), I/II/III/IV stages (2 studies), III stage (1 study) and IV stage (1 study). A TME was performed in all patients, and the type of rectal resection was reported in 24 studies (70.6%): anterior resection (23 studies), abdominoperineal resection (20 studies), Hartmann’s procedure (10 studies) and pelvic exenteration (3 studies).

#### Risk of bias

Seven domains for the potential risk of bias of included RCTs using methods described in the Cochrane Handbook for Systematic Reviews of Interventions were analysed [26]. All studies were rated as unclear risk of random sequence generation (selection bias) and five studies for allocation concealment (selection bias). Blinding of participants and personnel and incomplete outcome data were rated as high risk in all included studies. Five studies were rated as low risk of selection bias for selective reporting (reporting bias) and other bias. The ROBINS-I tool was used to evaluate the quality of the comparative studies.

### Primary outcomes

#### Local recurrence at 5 years

**Fig. 1:**
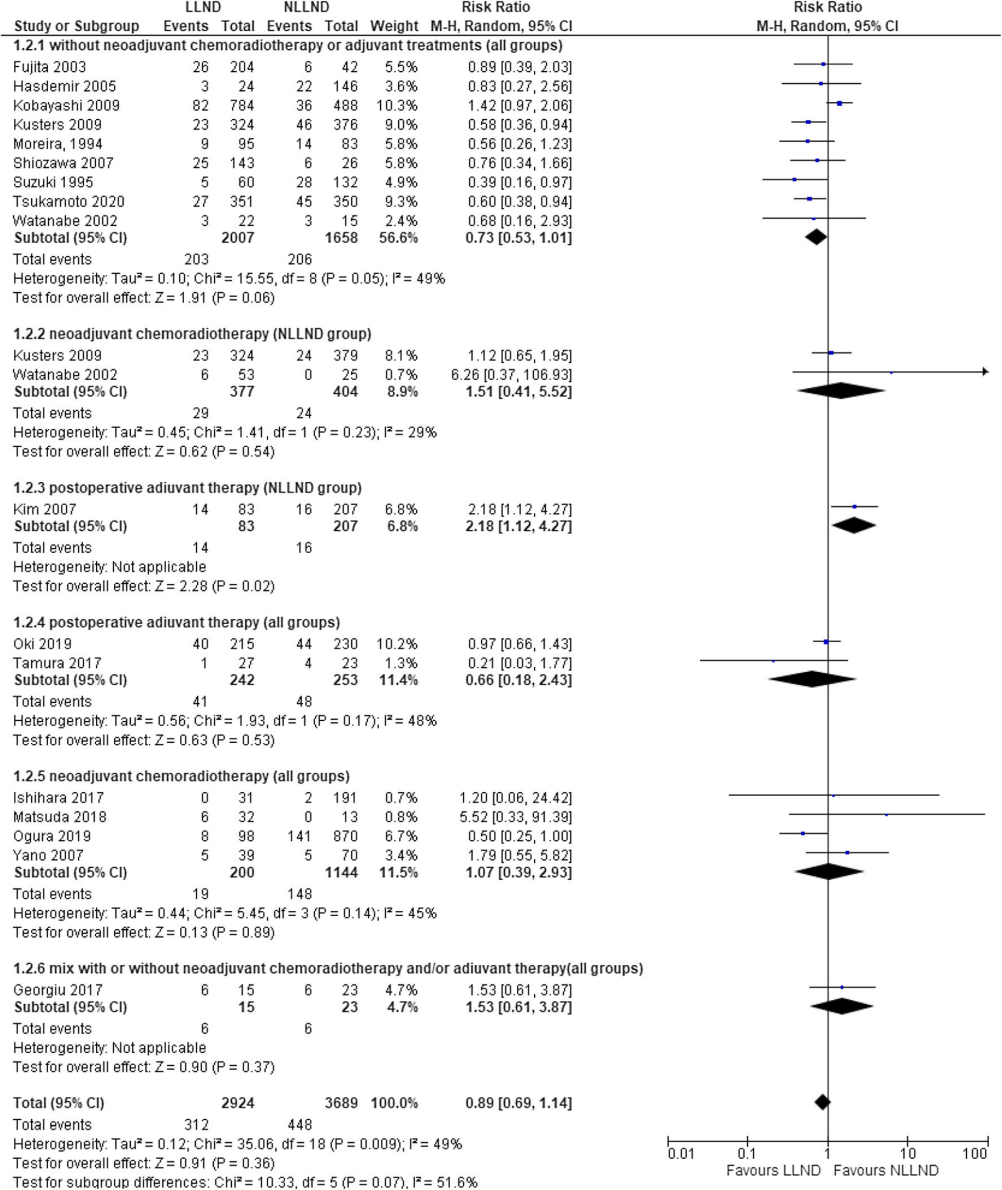
Forest plot, 5-year local recurrence

Seventeen studies [7, 17, 42, 45, 46, 48, 49, 52, 53, 57–61, 64, 66, 68, 69] reported local recurrence in 6613 patients (2.924 LLND and 3689 NLLND). The incidence of local recurrence was not statistically different between the overall LLND group (10.7%, 312/2.924) and the overall NLLND group (12.1%, 448/3.689) (RR 0.89, 95% CI 0.69 to 1.14; *I*^2^ = 49%, *P*=0.36) (Fig. 1).

In the subgroup analysis of patients who underwent LLND vs NLLND without (Fig. 1 (1.2.1)) or with adjuvant therapy (Fig. 1 (1.2.4)), there was no statistical difference between local recurrence rates in LLND (10.1%) and NLLND (12.4%) group [respectively RR 0.73 (95% CI 0.53 to 1.01) and RR 0.66 (95% CI 0.18 to 2.43)].

In the patients who underwent LLND vs NLLND with neoadjuvant CRT, local recurrence rate was the same in LLND and NLLND (RR 1.51, 95% CI 0.41–5.52) (Fig. 1 (1.2.2))

In all groups that underwent neoadjuvant CRT, there was not a significant difference in local recurrence rate in LLND group (RR 1.07, 95% CI 0.39–2.93) (Fig. 1 (1.2.5)).

#### Distant recurrence at 5 years

Four studies [7, 17, 45, 46, 60], including 888 patients (388 LLND and 500 NLLND), reported the rate of distant recurrence.

**Fig. 2:**
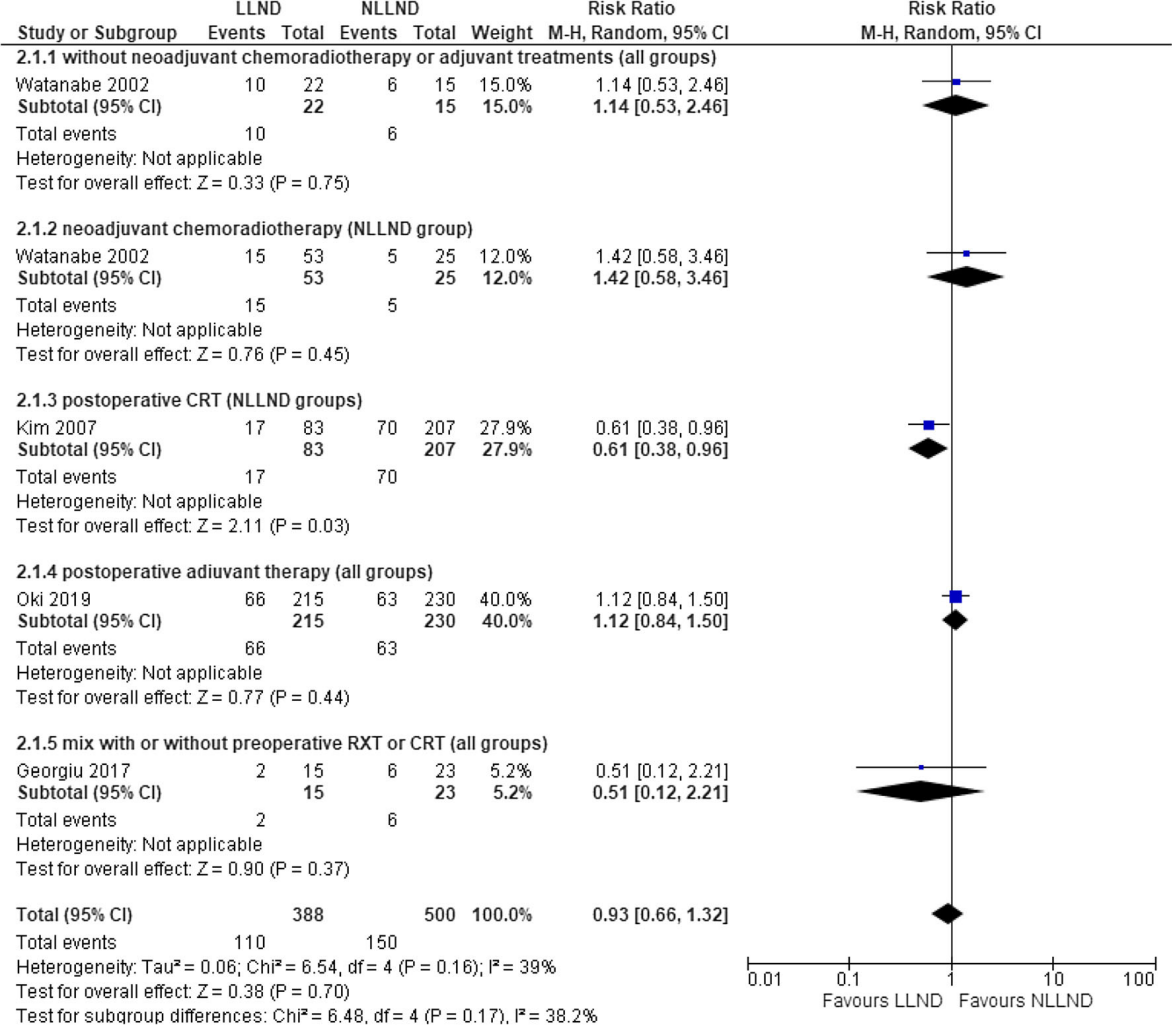
Forest plot 5-year distant recurrence

There was no significant difference in distant recurrence rate between the LLND group (28.6%, 110/388) and the NLLND group (30%, 150/500) (RR 0.93, 95% CI 0.66 to 1.32; *I*^2^ = 39%) (Fig. 2). In the subgroup analysis, the results did not show a statistically significant advantage for any group of patients, despite the better results in NLLND with neoadjuvant CRT compared with LLND alone (RR 1.42, 95% CI 0.58–3.46) (Fig. 2 (2.1.2)).

### Secondary outcomes

#### Overall 5-year survival

**Fig. 3:**
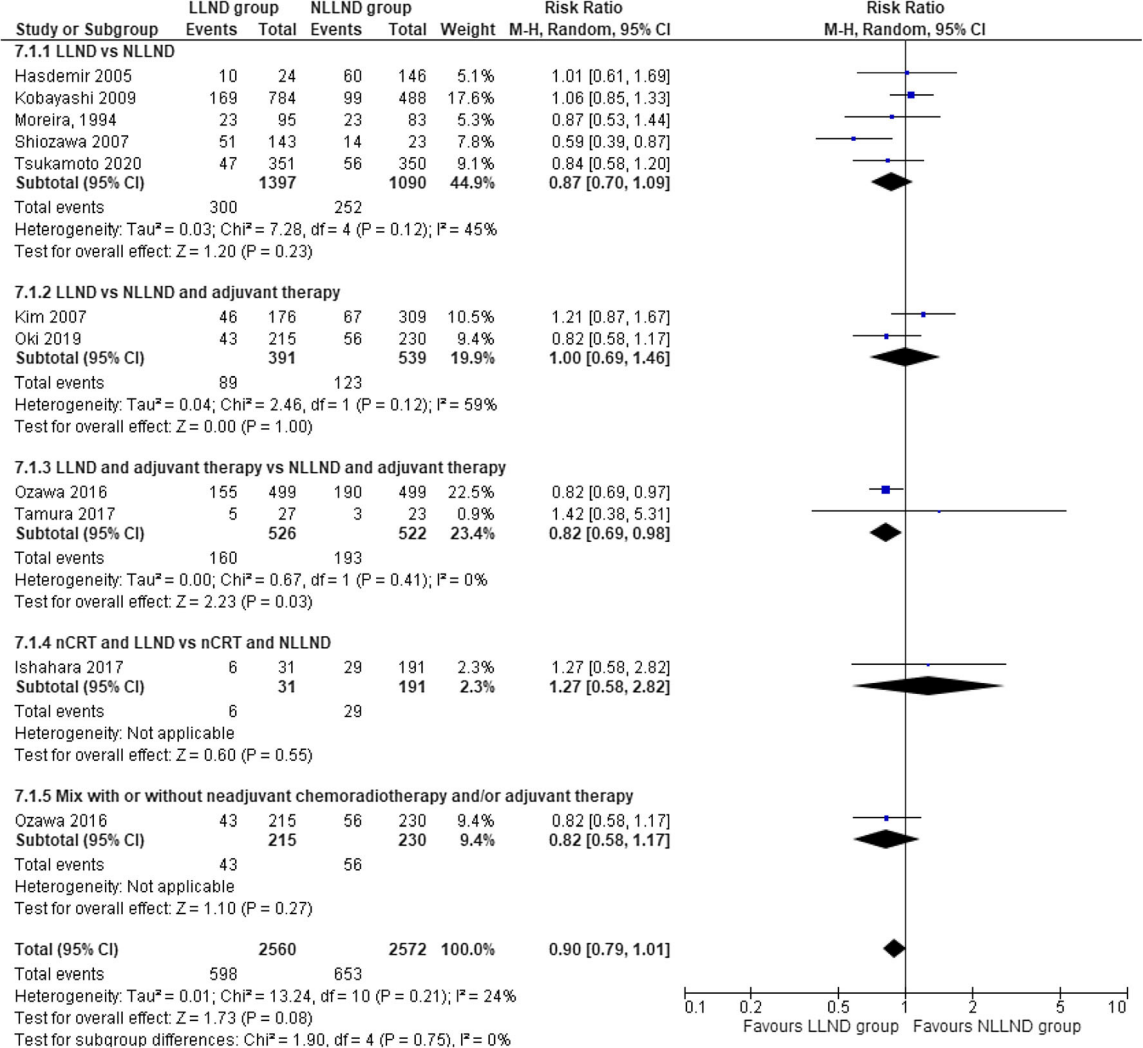
Forest plot overall survival at 5 years

Ten studies [41, 45, 46, 52, 53, 55, 58–60, 64, 69], including 5132 patients (2560 LLND and 2572 NLLND), reported the rate of this outcome. The overall survival at 5 years was not statistically different between the LLND group (76.6%) and the NLLND group (74.6%), (RR 0.90, 95% CI 0.79 to 1.01; *I*^2^ = 17%) (Fig. 3).

#### Disease-free 5-year survival

**Fig. 4:**
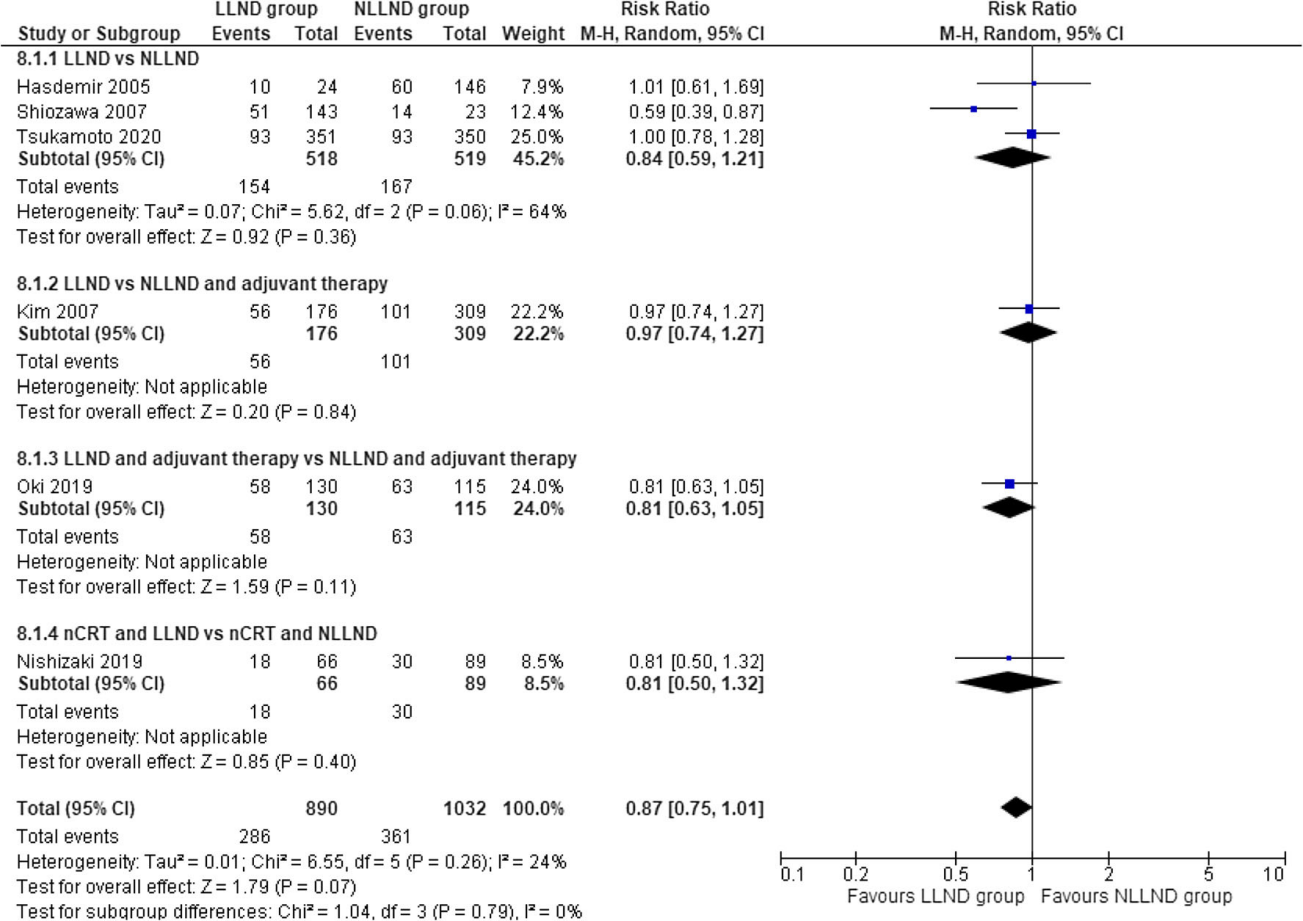
Forest plot disease-free survival at 5 years

Six studies (41, 45–47, 59, 60, 64), including 1922 patients (913 LLND and 1054 NLLND), reported the rate of this outcome. There was no statistical difference in terms of disease-free survival at 5 years when comparing the LLND group (67.9%) to the NLLND group (65%), (RR 0.87, 95% CI 0.75 to 1.01; *I*^2^ = 24%) (Fig. 4).

#### Urinary retention

**Fig. 5:**
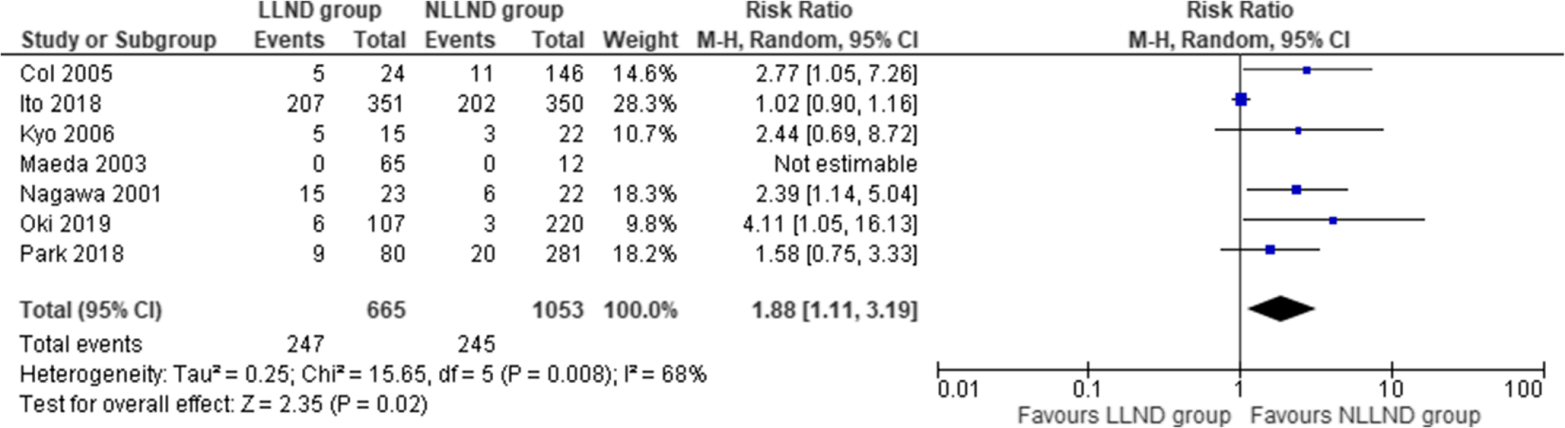
Forest plot urinary dysfunction (retention)

Seven studies [43, 45, 46, 50, 62, 63, 67], including 1718 patients (665 LLND and 1053 NLLND), reported urinary dysfunction. The incidence of urinary retention was significantly higher in the LLND patients (37%) if compared to the NLLND group (24.4%) (RR 1.88, 95% CI 1.11 to 3.19; *I*^2^ = 68%) (Fig. 5).

#### Urinary incontinence

**Fig. 6:**
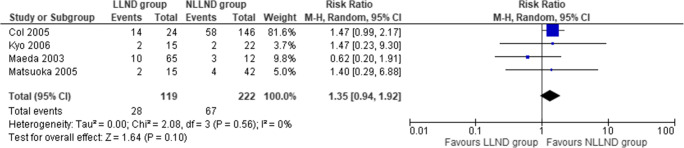
Forest plot urinary incontinence

Four studies [62, 63, 65, 67], including 341 patients (119 LLND and 222 NLLND), reported urinary incontinence. The incidence of urinary incontinence was similar between the LLND group (23.5%) and the NLLND group (27.4%) (RR 1.35, 95% CI 0.94 to 1.92; *I*^2^ = 0%) (Fig. 6).

#### Sexual dysfunction

**Fig. 7:**
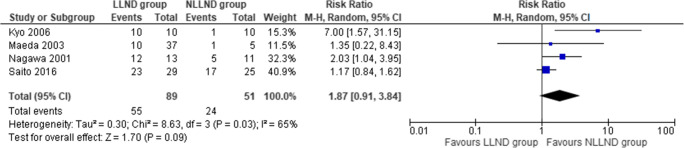
Forest plot sexual dysfunction

Four studies [44, 45, 62, 67], including 140 patients (27 LLND and 57 NLLND), reported sexual dysfunction. The incidence of sexual dysfunction was similar between the LLND group (61.7%) and the NLLND group (47%) (RR 1.87, 95% CI 0.91 to 3.84; *I*^2^ = 65%) (Fig. 7).

#### Evacuatory dysfunction

Two studies [45, 65], including 84 patients (27 LLND and 57 NLLND), reported evacuatory dysfunction. The incidence of evacuatory dysfunction was similar between the LLND group (62.9%) and the NLLND group (43.9%) (RR 1.57, 95% CI 1.00 to 2.47; *I*^2^ = 15%) (SDC 3)(ESM3).

## Discussion

Rectal cancer represents the third leading cause of death worldwide with a steadily increasing incidence [70, 71]. The concept of TME, introduced by Heald, has revolutionized the treatment by reducing the local recurrence rates from up to 40% to 4–8% [15, 71]. TME does not include the removal of the lateral pelvic lymph nodes but only those found within the mesorectal fascia and along the course of the mesenteric vessels. The efficacy of the excision of the pelvic lateral lymph nodes is still a controversial topic [72, 73].

**Fig. 8:**
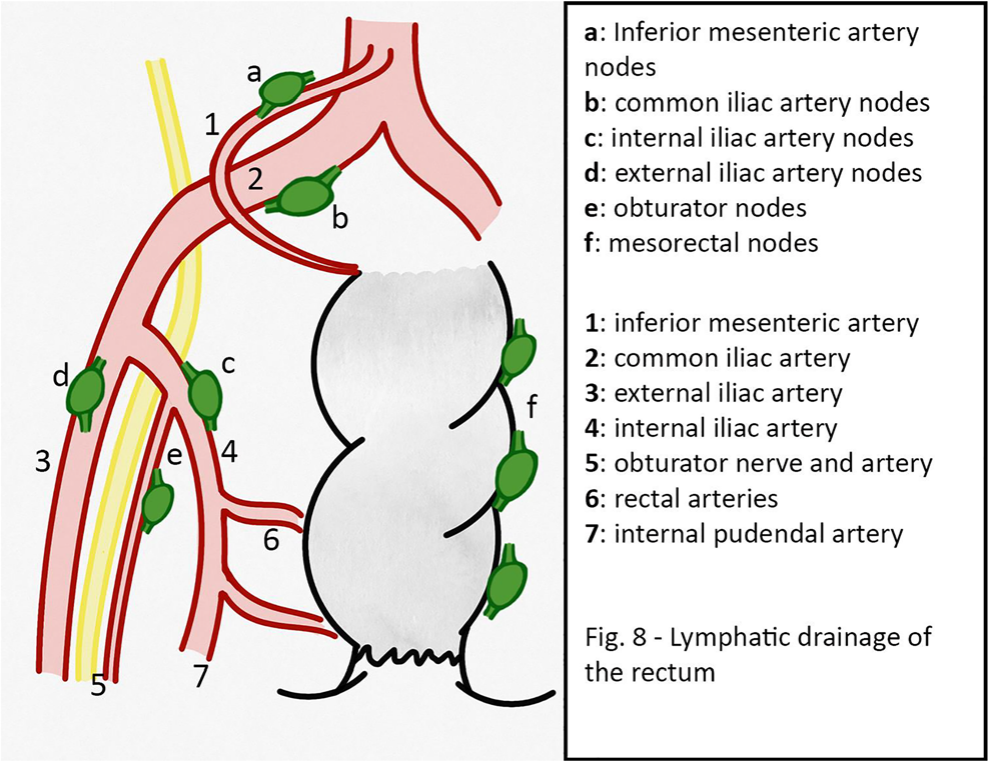
Lymphatic drainage of the rectum

The lymphatic drainage of the rectum, especially for the most distal/lower rectum, through the submucosal plexus, drains in three trunks: the upper branch, which flows into the lymphatic channels of the lower mesenteric vein; the middle branch, draining to the lymph nodes surrounding the internal, external and common iliac vessels; the lower branch draining to the inguinal lymph nodes [15] (Table 2; Fig. 8). These lateral regional lymphatic areas outside of the mesorectum are classified into six regions near the following arteries: the internal pudendal (outside of the pelvic plexus), the internal iliac (proximal to the superior vesical artery), the common iliac, the external iliac, the obturator and the presacral regions. Among these regions, the internal iliac artery and obturator regions have the highest rate of nodal involvement (22-61%) and are called the ‘vulnerable field’ [13, 14].

**Table 2:** Lymphatic drainage of the rectum

Lymphatic drainage	Lymph node group	Tributary Veins
Upper branch	Rectosigmoid nodes	Lower mesenteric vein
Middle branch	Iliac nodes Sacral nodes	Common iliac vein Internal Iliac vein External Iliac vein
Lower branch	Inguinal nodes Obturatory nodes External Iliac nodes Pelvic nodes	Epigastric vein Pudendal vein External iliac vein

The different approaches to the LLND between the East and West stems from the concept that pelvic lateral lymph nodes are considered regional according to Japanese authors and staging systems. The Western world, with the latest AJCC guidelines (AJCC 8^th^ edition), confirms pelvic lateral lymph node stations as remote stations. This is mainly debated in the case of stage II and III low rectal cancer. The involvement of lymph node stations in the iliac and obturator regions varies from 10.6 to 25.5% [15] stage II and III rectal cancer below the peritoneal reflection. More specifically, pelvic extra-regional lymph node involvement is reported in 5.4% of T1 cases, 8.2% for T2, 16.5% for T3 and 37.2% for T4 [58]. For this reason, Japanese surgeons suggest performing TME with bilateral pelvic lymphadenectomy without neoadjuvant treatment, as they expect that the risk of intrapelvic recurrence decreases by 50%, and 5-year survival improves by 8 to 9% [7, 8].

On the contrary, surgeons of the Western world generally treat rectal cancer with a classical TME and often considering neoadjuvant CRT [74], preserving LLND for patients with clinically suspected lateral pelvic lymph node metastasis [9–11].

The comparison between LLND versus CRT for lateral pelvic lymph nodes mainly concerns the rate of local pelvic recurrence. The only RCT comparing these two surgical techniques is JCOGO212 [41], which compared TME vs. TME and lateral pelvic lymphadenectomy in patients who had no lateral pelvic lymphadenopathy before surgery. The rate of local recurrence decreased from 12.6% in cases of TME alone to 7.4% when TME was associated with lateral lymphadenectomy. A limitation of this study was the choice of not performing preoperative CRT before TME, even when it would have been indicated according to Western guidelines [12, 74]. Long-term follow-up of JCOGO212 confirms the non-inferiority of TME alone compared to TME with pelvic lymphadenectomy in patients without clinically identifiable pelvic lymph node involvement. The study concludes that pelvic lateral lymphadenectomy should only be performed in patients with radiological evidence of lymph node involvement.

Other studies [54] confirm that the risk of pelvic recurrence rises to 19.5% in patients with lateral pelvic lymph nodes of a size more than 7 mm after neoadjuvant therapy. On the other hand, there is little evidence on the true efficacy of bilateral pelvic lymphadenectomy for low rectum carcinomas without clinical evidence of bilateral pelvic lymphadenopathy [41]. Although TME alone should not be considered inferior to TME with lateral lymphadenectomy, surgery extended to lateral pelvic lymph nodes reduces the risk of pelvic recurrence, especially in radiologically positive cases.

The main point of the discussion remains the risk of lateral pelvic lymph node metastases even after neoadjuvant CRT. The literature [72] reports a high percentage (up to 30–40%) of pelvic lymph node involvement even after neoadjuvant CRT.

The results from the present analysis confirm that the more radical and invasive surgical approach does not appear to be the safest and optimal way to treat these patients. The comparison between LLND and NLLND groups showed no difference in the rate of local recurrence and distant metastases. The central role in the prevention of local recurrences seems to be the use of neoadjuvant CRT, as the only group with statistically improved results was the non-LLND with neoadjuvant CRT when compared to LLND only. Regarding overall survival, the cumulative analysis also revealed a lack of any advantage of LLND, but the subgroup analysis did show improved overall survival in the group with LLND plus neoadjuvant CRT.

The main concern for the more invasive surgical approach of LLND is additional complications. It is recognized that higher occurrence of urinary, defecatory and sexual dysfunctions is found after LLND [3, 75], despite the introduction of nerve-sparing techniques. In the present analysis, the incidence of urinary retention and incontinence and sexual dysfunctions was directly compared in patients with and without LLND. The only statistically significant difference was the higher incidence of urinary retention in patients undergoing LLND. Another possible confounding factor is the fact that the comparison in most cases was carried out on patients without CRT, which is a procedure also burdened with similar and potentially additional, functional complications. More targeted studies are needed to assess the safety and quality of life following LLND surgery.

An important limitation of the present analysis is the possible bias introduced by the high heterogeneity of the clinical and oncological status of the included patients. Furthermore, our analysis could be expanded and completed by examining other data, such as the number of harvested lymph nodes, additional lymph node metastases detected in the LLND group and differences in functional outcomes between minimally invasive surgeries versus open resections.

## Conclusion

Our results suggest that TME with LLND does not offer an oncological advantage over TME without LLND. The advantage of LLND in the pre-neoadjuvant CRT era is lost after the implementation of neoadjuvant CRT. The addition of adjuvant CRT to LLND appears to contribute towards better survival and diminishes the rate of local recurrences. Whilst incurring in heterogeneity of data analysing currently available literature, the evidence would suggest that there is no place for routine LLND in the management of rectal cancer.

These findings reiterate the importance of careful selection of patients for LLND through an improved definition of pathological lymph nodes. Improved imaging techniques to accurately define a reliable cut-off size and describe radiological abnormalities that accurately predict involvement of pelvic lymph nodes are needed. Further studies, preferably prospective, that focus on survival and its association with surgical technique are needed to establish an evidence-based cut-off, which would aid in identifying precise indications for LLND.

## Supplementary information

ESM 1 PRISMA flow diagram (DOCX 33 kb)

ESM 2 Excluded studies (DOCX 19 kb)

ESM 3 Forest plot evacuatory dysfunction (DOCX 19 kb)

ESM 4 (DOCX 220 kb)

ESM 5 (PNG 3304 kb)

ESM 6 (DOC 64 kb)
