# Supplementary material for: Rise and fall of total mesorectal excision with lateral pelvic lymphadenectomy for rectal cancer: an updated systematic review and meta-analysis of 11,366 patients
Source: Int J Colorectal Dis. 2021 Jun 14;36(11):2321–33. doi: 10.1007/s00384-021-03946-2 (PMC8505280; doi:10.1007/s00384-021-03946-2)
Supplement: Supplementary file 1 — PRISMA flow diagram (DOCX 33 kb) [file 384_2021_3946_MOESM1_ESM.docx]

**SDC 1. Prisma flow chart of literature search**

Additional records identified through other sources (n= 9)

Records identified through database searching (n= 2.824)

Records after duplicates removed (n = 1.767)

Records excluded
(n = 1.724)

Records screened
(n = 1767)

Full-text articles excluded, with reasons SDC 1 (n = 9)

Full-text articles assessed for eligibility (n = 43)

Studies included in qualitative synthesis (n = 34)

Studies included in quantitative synthesis (meta-analysis)
(n = 28)
