# Supplementary material for: Rise and fall of total mesorectal excision with lateral pelvic lymphadenectomy for rectal cancer: an updated systematic review and meta-analysis of 11,366 patients
Source: Int J Colorectal Dis. 2021 Jun 14;36(11):2321–33. doi: 10.1007/s00384-021-03946-2 (PMC8505280; doi:10.1007/s00384-021-03946-2)
Supplement: Supplementary file 2 — Excluded studies (DOCX 19 kb) [file 384_2021_3946_MOESM2_ESM.docx]

**SDC 2 - Excluded studies and reason for exclusion**

| **Author – Year of Publication** | **Reason of exclusion** | **Systematic reviews in which the studies were included** | | | | | | | |
| --- | --- | --- | --- | --- | --- | --- | --- | --- | --- |
|  |  | **Hajibandeh**  **2020** | **Longchamp**  **2020** | **Gao**  **2020** | **Wang 2020** | **Law**  **2020** | **Ma**  **2020** | **Cheng 2011** | **Georgiou 2009** |
| Otowa 2015 (32) | LLND vs limited-LPLD in patients that underwent neoadjuvant chemoradiotherapy | No | No | No | No | Yes | No | No | No |
| Dong 2003 (33) | In the patients that underwent LLND, the Authors did not report rectal resection with TME | No | Yes | No | No | No | No | No | No |
| Shirouzu 2001 (34) | In the group of patients without LLND, the Authors included only patients who did not undergo TME | No | No | No | No | No | Yes | No | No |
| Havenga 1999 (35) | In the patients that underwent LLND, the Authors did not report rectal resection with TME | No | Yes | No | No | No | No | No | No |
| Michelassi 1993 (36) | The Authors reported a different technique for LLND: the pelvic lymphadenectomy included lymph nodes distal to the aortocaval bifurcation along the common and internal iliac vessels. | No | No | No | No | No | Yes | No | Yes |
| Hojo 1989 (37) | The Authors reported a different technique for LLND: en-bloc excision of pelvic lymph glands and adipose tissue of pelvic sidewall with removal of internal iliac vessels | No | No | No | No | No | Yes | No | Yes |
| Michelassi 1988 (38) | The Authors reported a different technique for LLND: the pelvic lymphadenectomy included lymph nodes distal to the aortocaval bifurcation along the common and internal iliac vessels | No | No | No | No | No | Yes | No | Yes |
| Enker 1986 (39) | The Authors reported a different technique of LLND: aortoiliac pelvic lymphadenectomy | No | No | No | No | No | Yes | No | Yes |
| Koyama 1984 (5) | The Authors reported a different technique for LLND: the pelvic lymphadenectomy included the removal of internal iliac vessels | No | No | No | No | No | No | No | Yes |

neoadjuvant chemoradiotherapy (nCRT)
