# Supplementary figures and images for: Rise and fall of total mesorectal excision with lateral pelvic lymphadenectomy for rectal cancer: an updated systematic review and meta-analysis of 11,366 patients
Source: Int J Colorectal Dis. 2021 Jun 14;36(11):2321–33. doi: 10.1007/s00384-021-03946-2 (PMC8505280; doi:10.1007/s00384-021-03946-2)

SDC 3. **Evacuatory dysfunction.**


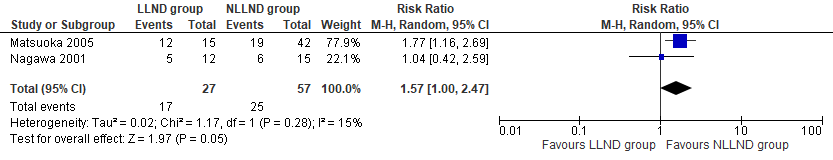

Supplement: Supplementary file 3 — Forest plot evacuatory dysfunction (DOCX 19 kb) [file 384_2021_3946_MOESM3_ESM.docx]
